# Behavioral interventions to reduce inappropriate antibiotic prescribing: a randomized pilot trial

**DOI:** 10.1186/s12879-016-1715-8

**Published:** 2016-08-05

**Authors:** Stephen D. Persell, Jason N. Doctor, Mark W. Friedberg, Daniella Meeker, Elisha Friesema, Andrew Cooper, Ajay Haryani, Dyanna L. Gregory, Craig R. Fox, Noah J. Goldstein, Jeffrey A. Linder

**Affiliations:** 1 Division of General Internal Medicine and Geriatrics, Feinberg School of Medicine, Northwestern University, 750 N. Lake Shore Drive, 10th Floor, Chicago, IL 60611 USA; 2 Center for Primary Care Innovation, Feinberg School of Medicine, Northwestern University, 750 N. Lake Shore Drive, 10th Floor, Chicago, IL 60611 USA; 3 USC Schaeffer Center for Health Policy and Economics, University of Southern California, 3335 S. Figueroa Street, Unit A, Los Angeles, CA 90089-7273 USA; 4 Division of General Internal Medicine and Primary Care, Brigham and Women’s Hospital; Harvard Medical School, 1620 Tremont Street, BC-3-2X, Boston, MA 02120 USA; 5 RAND, 20 Park Plaza, Suite 920, Boston, MA 02116 USA; 6 RAND, 1776 Main St., Santa Monica, CA 90401 USA; 7 Department of Psychology, UCLA Anderson School of Management; David Geffen School of Medicine at UCLA, UCLA, 110 Westwood Plaza D-511, Los Angeles, CA 90095 USA

**Keywords:** Antibiotics, Acute respiratory infections, Behavioral economics, Social psychology, Clinical decision support

## Abstract

**Background:**

Clinicians frequently prescribe antibiotics inappropriately for acute respiratory infections (ARIs). Our objective was to test information technology-enabled behavioral interventions to reduce inappropriate antibiotic prescribing for ARIs in a randomized controlled pilot test trial.

**Methods:**

Primary care clinicians were randomized in a 2 × 2 × 2 factorial experiment with 3 interventions: 1) Accountable Justifications; 2) Suggested Alternatives; and 3) Peer Comparison. Beforehand, participants completed an educational module. Measures included: rates of antibiotic prescribing for: non-antibiotic-appropriate ARI diagnoses, acute sinusitis/pharyngitis, all other diagnoses/symptoms of respiratory infection, and all three ARI categories combined.

**Results:**

We examined 3,276 visits in the pre-intervention year and 3,099 in the intervention year. The antibiotic prescribing rate fell for non-antibiotic-appropriate ARIs (24.7 % in the pre-intervention year to 5.2 % in the intervention year); sinusitis/pharyngitis (50.3 to 44.7 %); all other diagnoses/symptoms of respiratory infection (40.2 to 25.3 %); and all categories combined (38.7 to 24.2 %; all *p* < 0.001). There were no significant relationships between any intervention and antibiotic prescribing for non-antibiotic-appropriate ARI diagnoses or sinusitis/pharyngitis. Suggested Alternatives was associated with reduced antibiotic prescribing for other diagnoses or symptoms of respiratory infection (odds ratio [OR], 0.62; 95 % confidence interval [CI], 0.44–0.89) and for all ARI categories combined (OR, 0.72; 95 % CI, 0.54–0.96). Peer Comparison was associated with reduced prescribing for all ARI categories combined (OR, 0.73; 95 % CI, 0.53–0.995).

**Conclusions:**

We observed large reductions in antibiotic prescribing regardless of whether or not study participants received an intervention, suggesting an overriding Hawthorne effect or possibly clinician-to-clinician contamination. Low baseline inappropriate prescribing may have led to floor effects.

**Trial Registration:**

ClinicalTrials.gov: NCT01454960.

**Electronic supplementary material:**

The online version of this article (doi:10.1186/s12879-016-1715-8) contains supplementary material, which is available to authorized users.

## Background

Viruses cause the vast majority of acute respiratory infections (ARIs), yet antibiotics remain widely prescribed [1–3]. Inappropriate antibiotic use may lead to adverse drug events, greater cost, and the spread of resistant organisms [4–6].

Interventions to reduce antimicrobial prescribing such as physician and patient education, physician audit and feedback, clinical decision support and financial or regulatory incentives have been modestly effective at reducing inappropriate antibiotic prescribing, generally producing approximately 10 % reductions in antibiotic prescribing rates [7–9]. Educational interventions may have limited impact on prescribing rates when lack of guideline awareness is not the primary reason for inappropriate antibiotic prescribing [10].

Recognizing the limitations of educational and informational interventions, we developed several interventions that draw on insights from behavioral economics and social psychology [11]. These are designed to appeal to clinician self-image and social motivation. Our interventions take into account a growing body of research indicating that individuals’ cognitive limitations and social motivations often give rise to systematic biases that violate standards of rational behavior [12–15]. Here we report the findings of a single-site clinician-randomized controlled trial that was performed in preparation for a larger multi-site trial [16].

## Methods

### Objectives

The objective of the study was to pilot test three interventions to improve guideline-concordant antibiotic prescribing as a precursor to inform development of a larger national cluster randomized trial (ClinicalTrials.gov Identifier: NCT01454947) [11]. The Northwestern Medical Faculty Foundation (NMFF) General Internal Medicine clinic was selected as the pilot site prior to a larger trial conducted in three other organizations for two reasons: first, the NMFF site, with a single clinic, could not accommodate a cluster-randomized trial (with randomization at the clinic level); second, the NMFF site was able to start the trial earlier than the other sites, which permitted pilot testing of the chosen interventions and optimization of study procedures.

The institutional review boards of Northwestern University and the University of Southern California approved the study. The trial registration identifier is NCT01454960 at ClinicalTrials.gov. The study protocol is available from the corresponding author by request.

### Setting and participants

The NMFF General Internal Medicine clinic is a large adult primary care practice affiliated with an academic medical center and located in Chicago, Illinois. The practice uses the EpicCare electronic health record (EHR; Epic Systems Corp., Verona, WI) for all clinical documentation and for all prescribing. All attending physicians and nurse practitioners were eligible for recruitment (excluding SDP, a study investigator). Clinicians provided written informed consent before randomization. Payment for study participation was up to $300 per clinician per month of participation, regardless of intervention assignment (clinicians with less than full-time clinic work schedules were paid proportionally less). De-identified data from qualifying patient visits that occurred with enrolled clinicians were used in the analyses with an institutional review board waiver of consent.

### Baseline survey and education

On enrollment, we asked all participating clinicians to complete a 15 to 20 min online survey that included questions about attitudes regarding antibiotic prescribing and an educational module containing information about ARI treatment guidelines [17–20]. The module also described the interventions to which the clinician was assigned, including changes they would observe in their EHR (for Accountable Justifications and Suggested Alternatives interventions) and examples of the kinds of emails they would receive (Peer Comparison). Brief descriptions of the interventions are provided below; details are available elsewhere [11].

### Interventions

#### Accountable justifications intervention

Clinicians randomized to “Accountable Justifications” received EHR alerts in the course of e-prescribing an antibiotic for an ARI diagnosis (listed in the Additional file 1: Table S1). The alert briefly summarized the treatment guidelines corresponding to the ARI diagnosis for which the antibiotic was being written (e.g., “antibiotics are not indicated for non-specific upper respiratory infections”), prompted the clinician to enter a free-text justification for prescribing an antibiotic, and informed the clinician that the free-text justification provided would be included in the patient’s medical record where it would be visible to other clinicians. Clinicians were also informed that if no free-text justification was entered, a default statement “No justification for prescribing antibiotics was given” would appear in the record. If the antibiotic order was canceled, no justification was required, and no default text appeared.

These alerts were suppressed for patients whose EHR problem lists contained comorbid chronic conditions that exempted these patients from clinical guidelines (e.g. chronic obstructive pulmonary disease, heart failure, cirrhosis). The lists of diagnoses we used to suppress study-related clinical decision support were similar to those published in the supplement to the article describing the methods for the multi-site study [11].

This intervention draws from prior studies that show accountability improves decision making accuracy, and that public justification engenders reputational concerns. We expected that the potential to have their non-guideline-concordant choices displayed to others would make clinicians more likely to act in accordance with injunctive norms (guideline recommendations) [21–26].

#### Suggested alternatives intervention

When clinicians assigned to the Suggested Alternatives intervention entered an ARI diagnosis for a patient visit, a computerized alert presented an order set containing multiple non-antibiotic prescription and non-prescription medication choices as well as educational materials that could be printed and given to the patient [11]. We designed these order sets to include many of the most common non-antibiotic treatments used to treat ARI symptoms. This intervention draws from the behavioral insight that the presentation of (non-antibiotic) alternatives may lead clinicians to infer that these suggestions ought to be considered and this will lead to a reduced chance that an antibiotic will be prescribed [27].

#### Peer comparison intervention

Clinicians in the Peer Comparison Intervention group received emailed monthly performance feedback reports. These reports included the clinician’s individual antibiotic prescribing rates for non-antibiotic-appropriate ARIs and as a benchmark, the antibiotic prescribing rate for clinicians who were at the 10th percentile within the clinic (i.e., those with the lowest rates of inappropriate antibiotic prescribing). These rates were calculated based on the most recent 20 eligible visits excluding encounters occurring with patients who had certain comorbidities or other diagnosed bacterial infections [11].

If clinicians were among the 10 % of their peers with the “best”—i.e. lowest— prescribing rates the emailed reports told clinicians "You are a top performer.” If clinicians were not among the 10 % best, the emailed report told clinicians “You are not a top performer. You are prescribing too many unnecessary antibiotics.” The proportion of “Top Performers” could be greater than 10 % of clinicians if more than 10 % of clinicians had an inappropriate antibiotic prescribing rate of zero.

These peer comparisons were designed to differ from traditional audit and feedback by showing comparisons to top-performing instead of average-performing peers, and its delivery of positive reinforcement to current top-performers. This strategy has been previously shown to help sustain high performance [28–30].

### Experimental design and randomization

We performed a 2 × 2 × 2 factorial randomized trial, with one arm for each possible combination of the three interventions, corresponding to the eight rows shown in Table 2. We randomized all clinicians at once in two blocks by number of qualifying visits in the prior year to ensure relatively balanced allocation, then assigned each to an intervention group using a random number generator in SAS 9.3 (SAS Institute Inc., Cary, NC) performed by a researcher who was not aware of clinicians’ identities until after the randomization was completed [31]. Once the intervention began, participating clinicians were not blinded to their study group assignments. Outcome data was collected and assessed automatically (with no human judgement) by applying identical criteria to data collected using Structured Query Language applied to EHR data.

### Measures

We measured physician and nurse practitioner characteristics during the initial online survey. We obtained patient age, sex and race/ethnicity from EHR data.

#### Primary outcome

The primary study outcome was the rate of oral antibiotic prescribing during eligible study visits with non-antibiotic-appropriate ARI diagnoses (listed Additional file 1: Table S1). An office visit was eligible for inclusion in the outcome denominator if: 1) the patient was 18 years old or older, 2) the clinician was enrolled in the study, 3) the visit occurred during the 12-month intervention period from February 2012 through January, 2013, and 4) the patient did not have a visit with an ARI diagnosis in the prior 30 days. We excluded visits from the primary analysis when: 1) patients had certain medical co-morbidities that make ARI guidelines less likely to apply; 2) patients had concomitant visit diagnoses indicating a possible non-ARI bacterial infection or reason for antibiotic prescribing; or 3) patients had concomitant visit diagnoses indicating potentially antibiotic-appropriate ARI diagnoses or other ARI diagnoses suggestive of a bacterial infection. The sets of diagnoses used to determine eligibility and calculate the outcomes were similar to those we reported previously [11].

#### Secondary outcomes

Secondary outcomes included the rates of oral antibiotic prescribing for three other groups of qualifying visits: 1) visits with diagnoses for potentially-antibiotic-appropriate ARI diagnoses (acute sinusitis and acute pharyngitis; Additional file 1: Table S1); 2) visits with diagnoses also potentially indicating an ARI (e.g., pneumonia, cough; Additional file 1: Table S1); and 3) the rate of antibiotic prescribing for the sum of visits included in the primary outcome and these two secondary outcomes (all diagnosis categories combined). We applied the same additional inclusion and exclusion criteria above to these secondary diagnoses as we did to the primary outcome. We examined the distribution of visits in each of these 3 categories during the intervention year and the year prior to look for “diagnosis shifting” through which clinicians could be more likely to select antibiotic-appropriate diagnoses to conceal an unchanged overall antibiotic prescribing rate.

#### Safety monitoring

In order to ensure that interventions did not cause inappropriate under-prescribing of antibiotics, we examined the records of all patients who had return visits with a diagnosis indicative of a bacterial respiratory tract infection within 30 days of a qualifying visit for a non-antibiotic-appropriate diagnosis, a potentially-antibiotic-appropriate diagnosis, or cough. In cases where an antibiotic was not prescribed at the index visit, a physician (SDP) blinded to clinician intervention group judged whether: 1) an antibiotic was prescribed within 24 h without the patient having to reinitiate contact (e.g. physician called the patient and prescribed an antibiotic in response to an abnormal chest x-ray), 2) an antibiotic was not prescribed and it seemed unlikely that an antibiotic at the index visit would have improved the clinical course, or 3) an antibiotic was not prescribed and it may have been clinically useful had an antibiotic been prescribed. An independent data safety and monitoring board reviewed this data during the study.

### Statistical analysis

The effects of the study interventions were assessed using separate mixed logistic regression models for each of the primary and secondary outcomes that included each of the three interventions and clinicians’ prior prescribing rate as fixed effects, and individual clinicians as random effects (PROC GLIMMIX, SAS 9.3). We tested models with and without two-way and three-way interaction terms between the interventions. Because including these interaction terms had very little impact on the estimated effects of the interventions and complicate the interpretation of the results, we report results of models without interaction terms. Because this was intended as a pilot study, no formal power analysis was conducted. Results are provided with 95 % confidence intervals.

In addition to the primary planned analyses, post hoc we constructed models for each of the primary and secondary outcomes that included eligible visits during the year prior to and the year during the intervention period. We tested models that included a term for the intervention year and also a continuous variable for time to assess for evidence of temporal changes in antibiotic prescribing rates.

We performed post hoc analyses that included data from the 10 physicians working in the same practice who did not participate in the randomized trial during the year prior to and the year during the intervention period to determine if their antibiotic prescribing rates during these two years differed significantly from the clinicians who participated in the study.

## Results

Of the 37 internists and 1 nurse practitioner approached, 27 internists and the nurse practitioner enrolled in the trial (74 %). Participating clinicians were 39 male, 61 white, 36 Asian and 4 % African American. Most (75 %) were over 40 years of age. The average number (standard deviation) of distinct patients seen by a participating clinician was 905 (483) in the pre-intervention year and 923 (543) in the intervention year. The flow of participants through the trial is shown in the Additional file 2: Figure S1. Of all visits made to participating clinicians in the pre-intervention year, 7.2 % were eligible ARI visits analyzed for any of the study outcomes. In the intervention year 7.7 % were eligible ARI visits. Patients’ demographic characteristics are provided in Table 1.

**Table 1:** Demographic characteristics of patients seen by participating clinicians

	All Patients		Patients Included in Study Analyses	
	Pre-intervention	Intervention	Pre-intervention	Intervention
	Year	Year	Year	Year
Age, mean (SD)	48.7 (16.7)	49.4 (16.9)	46.6 (16.2)	47.7 (16.3)
Sex, n (%)				
Male	7269 (36.2)	7469 (36.6)	904 (32.2)	888 (33.1)
Female	12798 (63.8)	12950 (63.4)	1907 (67.8)	1791 (66.9)
Race/Ethnicity, n (%)				
White, non-Hispanic	9460 (47.1)	9659 (47.3)	1384 (49.2)	1244 (46.4)
Black	3706 (18.5)	3806 (18.6)	422 (15)	444 (16.6)
Hispanic	1380 (6.9)	1457 (7.1)	201 (7.2)	204 (7.6)
Asian	974 (4.9)	1056 (5.2)	121 (4.3)	129 (4.8)
American Indian/Pacific Islander	42 (0.2)	50 (0.2)	11 (0.4)	9 (0.3)
More than one race	359 (1.8)	414 (2)	52 (1.8)	58 (2.2)
Other	1481 (7.4)	1524 (7.5)	213 (7.6)	225 (8.4)
Unknown	2665 (13.3)	2453 (12)	407 (14.5)	366 (13.7)

The number of clinicians randomized to the different intervention assignments is shown in Table 2 along with the number of eligible study visits with a non-antibiotic-appropriate ARI diagnosis during the year prior to the study and the year during the study. Additional details of are provided in the study flow diagram (Additional file 2: Figure S1). Participating physicians prescribed antibiotics in 24.7 % of visits for non-antibiotic-appropriate ARI diagnoses in the year prior to the trial and in 5.2 % of such visits during the year of the trial (Table 2). Corresponding results for the secondary outcomes are provided in Additional file 1: TablesS2, S3 and S4. None of the three interventions significantly lowered (or raised) rates of antibiotic prescribing for visits with non-antibiotic-appropriate diagnoses (Table 3). However, for visits with other ARI diagnoses and symptoms, the Suggested Alternatives intervention was associated with significantly lower odds of antibiotic prescribing (odds ratio [OR], 0.62; 95 % confidence interval [CI], 0.44–0.89). The Suggested Alternatives and Peer Comparisons interventions were associated with lower odds of antibiotic prescribing for the all ARI diagnoses combined—OR 0.72 (CI 0.54–0.96) and OR 0.73 (CI 0.53–0.995), respectively. The clinician’s prescribing rate in the pre-trial year was strongly associated with the antibiotic prescribing rate during the trial year for each outcome examined (Table 3).

**Table 2:** Results of clinician randomization and the primary outcome

Intervention	Randomized Clinicians (n)	Antibiotic Prescribing for Visits with Non-Antibiotic-Appropriate ARI Diagnoses		Difference in Antibiotic Prescribing Rate between Intervention and Pre-intervention Period % (95 % CI)
		Pre-Intervention Year, n/N (%)	Intervention Year, n/N (%)	
No intervention	4	7 / 57 (12.3)	5 / 136 (3.4)	−8.6 (−17.9 to 0.7)
Accountable Justifications	3	14 / 72 (19.4)	8 / 70 (11.4)	−8.0 (−20.0 to 4.0)
Suggested Alternatives	3	7 / 44 (15.9)	4 / 51 (7.8)	−8.1 (−21.5 to 5.4)
Peer Comparisons	3	31 / 74 (41.9)	8 / 68 (11.8)	−30.1 (−44.0 to −16.3)
Accountable Justifications, Suggested Alternatives	4	20 / 128 (15.6)	2 / 95 (2.1)	−13.5 (−20.5 to −6.5)
Suggested Alternatives, Peer Comparisons	4	41 / 118 (34.8)	4 / 102 (3.9)	−30.8 (−40.3 to −21.3)
Accountable Justifications, Peer Comparisons	4	44 / 187 (23.5)	6 / 206 (2.9)	−20.6 (−27.2 to −14.1)
Accountable Justifications, Suggested Alternatives, Peer Comparisons	3	46 / 171 (26.9)	9 / 162 (5.6)	−21.4 (−28.9 to −13.8)
Any Accountable Justifications	14	124 / 558 (22.2)	25 / 532 (4.7)	−17.5 (−21.4 to −13.6)
No Accountable Justifications	14	86 / 293 (29.4)	21 / 357 (5.9)	−23.5 (−29.3 to −17.7)
Any Suggested Alternatives	14	114 / 461 (24.7)	19 / 410 (4.6)	−20.1 (−24.5 to −15.7)
No Suggested Alternatives	14	96 / 390 (24.6)	27 / 479 (5.6)	−19.0 (−23.7 to −14.2)
Any Peer Comparisons	14	162 / 550 (29.5)	27 / 537 (5.0)	−24.4 (−28.7 to −20.2)
No Peer Comparisons	14	48 / 301 (15.9)	19 / 352 (5.4)	−10.6 (−15.3 to −5.8)
All groups combined	28	210 / 851 (24.7)	46 / 889 (5.2)	−19.5 (−22.8 to −16.3)

*ARI* acute respiratory infection, *CI* confidence interval

**Table 3:** Intervention effects on primary and secondary outcomes

	Accountable justifications	Suggested alternatives	Peer comparisons	Clinician’s prior year prescribing rate (per 10 % increase)
	Odd ratios (95 % confidence intervals) for antibiotic prescribing			
Antibiotic for non-antibiotic appropriate ARI diagnoses (primary outcome)	0.98 (0.42–2.29)	0.68 (0.29–1.58)	0.45 (0.18–1.11)	1.57 (1.15–2.13)^*^
Antibiotic for potentially antibiotic appropriate ARI diagnoses	0.77 (0.42–1.41)	0.57 (0.31–1.05)	1.14 (0.59–2.19)	1.71 (1.23–2.36)^*^
Antibiotic for other ARIs diagnoses or symptoms of interest	1.29 (0.92–1.80)	0.62 (0.44–0.89)^**^	0.70 (0.48–1.02)	1.40 (1.25–1.57)^**^
Antibiotic for all three combined	1.05 (0.80–1.39)	0.72 (0.54–0.96)^***^	0.73 (0.53–0.995)^****^	1.64 (1.45–1.84)^***^

*ARI* acute respiratory infection

^*^ *p* < .005

^**^ *p* < .01

^***^ *p* < .001

^****^ *p* < 0.05

Table 4 shows the distribution of eligible visits among the three different diagnosis categories we examined in the pre-trial year and the trial year. There were similar total numbers of eligible study visits in each year (3276 in the pre-trial year and 3099 in the trial year). There was no suggestion of diagnosis shifting in the selection of diagnosis codes during the study.

**Table 4:** Antibiotic prescribing for eligible visits with all potential ARI diagnoses, pre-intervention year and intervention year

Diagnosis Category	Year prior to intervention				Intervention year			
	% of ARI visits in diagnosis category	Number visits given antibiotic	Number eligible visits	Percentage prescribed antibiotic	% of ARI visits in diagnosis category	Number visits given antibiotic	Number eligible visits	Percentage prescribed antibiotic
Non-Antibiotic Appropriate ARI Diagnoses	26.0	210	851	24.7	28.7	46	889	5.2
Potentially Antibiotic Appropriate ARI Diagnoses	24.9	411	817	50.3	24.3	336	752	44.7
Other ARIs Diagnoses or Symptoms of Interest	49.1	646	1608	40.2	47.0	369	1458	25.3
All diagnoses combined	100	1267	3276	38.7	100	751	3099	24.2

*ARI* acute respiratory infection

**Fig. 1:**
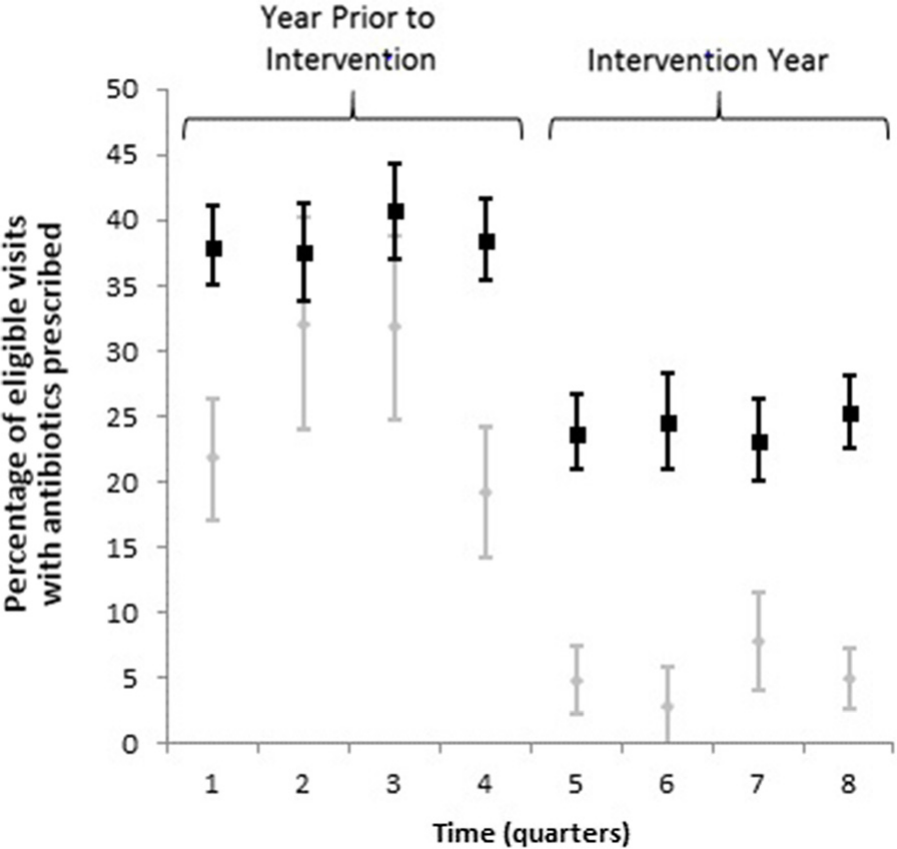
Percentage of eligible visits with antibiotic prescribed for visits with non-antibiotic appropriate diagnoses and all ari diagnosis categories combined by quarter. (Black Square) All eligible visits for acute respiratory infections. (Gray Diamond) Eligible visits for non-antibiotic-appropriate acute respiratory infections. Error bars represent 95 % confidence intervals. ARI: acute respiratory infection

In the mixed regression models, antibiotic prescribing was substantially and significantly lower during the intervention year compared to the pre-intervention year across all categories, and the continuous variable for time (study quarter, used to assess for an underlying temporal trend in antibiotic prescribing) was not significant in any of the four models examined except for a slight increase in prescribing rate over time for possibly-antibiotic-appropriate diagnoses (odds ratio [OR] per calendar quarter: 1.11 [1.03–1.20]; Table 5; Fig. 1).

**Table 5:** Results of mixed effects models pre-intervention year and intervention year^a^

	Year prior to the intervention	Calendar quarter
	Odds Ratios (95 % confidence Intervals)	
Antibiotic for non-antibiotic appropriate ARI diagnoses	6.34 (3.49–11.5)^*^	1.01 (0.90–1.14)
Antibiotic for potentially-antibiotic-appropriate ARI diagnoses	2.40 (1.69–3.42)^*^	1.11 (1.03–1.20)^**^
Antibiotic for other ARIs diagnoses or symptoms of interest	1.59 (1.10–2.29)^***^	0.98 (0.90–1.06)
Antibiotic for all three combined	2.28 (1.83–2.83)^*^	1.03 (0.98–1.08)

*ARI* acute respiratory infection

^*^ *p* < 0.001

^**^ *p* <0.01

^**^ *p* < 0.05

^a^Models predicting antibiotic prescribing include a pre-intervention year variable and a continuous variable for calendar quarter as fixed effects, and clinician as random effects. Analyses are exploratory

In the post hoc analysis, non-participating physicians prescribed antibiotics in 35.3 % of visits for non-antibiotic-appropriate ARI diagnoses in the year prior to the trial and in 28.6 % of such visits during the year of the trial. For all ARI diagnoses combined, prescribing rates for non-participants during these two years were 49.6 % and 46.2 %, respectively. In multivariable regression models, non-participants’ prescribing rates were not significantly different from those of study participants during the year prior to the intervention but were significantly higher than study participants during the trial period. The OR for prescribing antibiotics for non-antibiotic-appropriate ARI visits during the trial period for non-participants compared to participants was 4.99 (2.35–10.6), and for all ARI diagnoses combined OR 1.70 (1.31–2.21).

In the safety monitoring analyses, we analyzed 18 return visits (0.74 %) with a diagnosis of a bacterial respiratory tract infection that occurred within 30 days of a qualifying study visit at which an antibiotic was not prescribed. Of these, 7 of these received antibiotics within 24 h of the qualifying visit without the patient having to reinitiate contact. In 3 cases, it seemed unlikely that an antibiotic would have improved the course of illness if prescribed at the initial visit. In 8 cases (0.31 %), an antibiotic was not prescribed and may have been clinically useful had it been prescribed at the index visit. None of these 8 patients died or had a prolonged hospitalization. These visits were evenly distributed across the intervention groups (Additional file 1: Table S5).

## Discussion

This pilot study tested three interventions drawing on social psychology and behavioral economic principles. This initial test of these interventions helped inform the conduct of a larger, multi-site trial [16], and also led to several interesting observations. The study resulted in large reductions in antibiotic prescribing at adult outpatient visits for acute respiratory infections in a large primary care practice compared to the year before the study began. Reductions in antibiotic prescribing occurred both for clinicians receiving one or more active interventions and those assigned to the control condition (but not among physicians who declined to participate). The decline in prescribing occurred in all diagnosis categories examined and was most dramatic for visits with non-antibiotic-appropriate diagnoses (a decline of 19.5 %). During the intervention year, receipt of our interventions did not significantly affect antibiotic prescribing at visits with non-antibiotic-appropriate diagnoses. Two of the interventions, Suggested Alternatives and Peer Comparisons, yielded significantly lower rates of antibiotic prescribing for the secondary outcome of all the diagnostic categories combined. Compared to our larger, clinic-randomized trial, the overall reduction in antibiotic prescribing observed here was similar. Notable differences between that study and the one presented here are that in the larger study—which had greater statistical power—accountable justifications and peer comparisons led to significantly greater reductions in antibiotic prescribing at non-antibiotic-appropriate ARI visits [16].

The magnitude of the reduction in antibiotic prescribing observed from the pre-study year to the study year was larger than what has generally been observed in prior studies aimed at reducing antibiotic prescribing in ARIs. For instance, Gonzales and colleagues recently observed 12 to 13 % declines in antibiotic prescribing for acute bronchitis at intervention sites given computerized or printed decision support for acute cough illnesses and a small increase in antibiotic prescribing at control sites [9]. Likewise, a systematic review of quality improvement interventions aimed at reducing inappropriate antibiotic use showed a median reduction in antibiotic prescribing of 9.7 % (interquartile range 6.6—13.7 %) [7].

There are several possible explanations for our observed findings of a large overall decline in prescribing with little observable intervention-specific effects. First, clinicians’ enrollment in the study, completion of the on-line educational module, receipt of a financial incentive to participate in the study, and awareness that they were among a small group having their antibiotic prescribing practices scrutinized may have given rise to a fairly strong observer effect (or Hawthorne effect) even among clinicians assigned to the control condition [32]. This phenomenon has been described among pediatricians who had their antibiotic prescribing behavior observed [33]. In fact, we expected the Peer Comparison and Accountable Justifications interventions to operate partly through participants’ sensitivity to being observed by others. Physicians within this practice were accustomed to having their performance measured for a variety of clinical topics for quality improvement purposes [34], and the observer effect may have been particularly strong. Because the antibiotic prescribing rate among this clinician group prior to the intervention year was already lower than what has been reported in other studies [1, 2, 7], the prescribing rate for the primary outcome may have reached a floor so that adding additional interventions could not reduce it further.

Second, contamination from working in close proximity to peers who received single or multiple interventions related to antibiotic prescribing may have led clinicians to reduce their prescribing more than they otherwise would have. For example, learning that his colleague was a “top performer” relative to a group that includes his own performance may have instilled a sense of competition in a control participant. Such informational contamination between conditions could have been due to information concerning interventions, information about antibiotic prescription rates and/or general conversation about antibiotic over-prescribing. The fact that prescribing rates among non-participating physicians within the same physical practice changed little and remained substantially greater than rates for study participants speaks against this possibility.

Third, this study is limited by its sample size. This was a fairly small pilot study meant to test procedures to be employed in a larger multisite study [11]. The number of eligible visits for the primary outcome was insufficient to exclude clinically meaningful intervention effects on the primary study outcome. Furthermore, because we were testing the factorial design that was to be used in the subsequent larger trial, there were only three or four clinicians within each intervention assignment and only four clinicians who received none of the interventions. The small sample sizes probably account for highly variable baseline antibiotic prescribing rates between groups. Despite lack of statistical significance, we observed the expected direction of effects, and the magnitude of effects in the Suggested Alternatives and Peer Comparison interventions are promising.

Consistent with prior studies [10, 35, 36], we did not observe any evidence that clinicians manipulated their diagnostic coding away from non-antibiotic-appropriate diagnoses toward those that would appear to more readily justify the use of antibiotic or those that would not trigger the clinical decision support interventions. This provides reassurance that the changes in antibiotic prescribing rates that we observed were real, and not merely caused by changes in diagnosis selection.

We also observed a reduction in antibiotic prescribing at potentially-antibiotic-appropriate ARI visits (which consist of acute sinusitis and pharyngitis). These clinical syndromes are frequently caused by viruses, and our safety analysis showed very few patients returning within 30 days following a visit where an antibiotic was not prescribed and it may have been clinically useful had one been given. Therefore, we think it is likely that the reduction in antibiotic use in this diagnostic category was clinically appropriate.

An additional potential limitation is worth noting. The baseline antibiotic prescribing rates among clinicians in this group (both among clinicians who participated and those who didn’t) were lower than what has been reported in broader populations of clinicians [1–3]. This suggests that these findings may not be generalizable to other practice settings.

## Conclusions

The conduct of this pilot study had a large impact on antibiotic prescribing across all study physicians including among control participants (but not among non-participants within the same practice). The results of a larger multisite trial conducted by this same study group help address this study’s aforementioned limitations [16]. The large reductions in antibiotic prescribing we observed even among control participants are interesting and suggest that interventions designed with an intentional observer effect could be particularly effective.

## Abbreviations

ARI, acute respiratory infection; CI, confidence interval; EHR, electronic health record; NMFF, Northwestern Medical Faculty Foundation; OR, odds ratio

## Additional files

Additional file 1:
**Table S1.** Acute Respiratory Infection Diagnoses Related to Interventions and Outcomes Assessments. **Table S2.** Results of Clinician Randomization and the Secondary Outcome Potentially-Antibiotic-Appropriate ARI Diagnoses. **Table S3.** Results of Clinician Randomization and the Secondary Outcome Other ARI Diagnoses or Symptoms of Interest. **Table S4.** Results of Clinician Randomization and the Secondary Outcome All ARI Categories Combined. **Table S5.** Distribution of Safety Monitoring Events. (DOCX 27 kb) Additional file 2: Study Flow Diagram. (PPTX 77 kb)
