# Supplementary material for: Behavioral interventions to reduce inappropriate antibiotic prescribing: a randomized pilot trial
Source: BMC Infect Dis. 2016 Aug 5;16:373. doi: 10.1186/s12879-016-1715-8 (PMC4975897; doi:10.1186/s12879-016-1715-8)
Supplement: Additional file 1: — Table S1. Acute Respiratory Infection Diagnoses Related to Interventions and Outcomes Assessments. Table S2. Results of Clinician Randomization and the Secondary Outcome Potentially-Antibiotic-Appropriate ARI Diagnoses. Table S3. Results of Clinician Randomization and the Secondary Outcome Other ARI Diagnoses or Symptoms of Interest. Table S4. Results of Clinician Randomization and the Secondary Outcome All ARI Categories Combined. Table S5. Distribution of Safety Monitoring Events. (DOCX 27 kb) [file 12879_2016_1715_MOESM1_ESM.docx]

**Additional file 1: Table S1. Acute Respiratory Infection Diagnoses Related to Interventions and Outcomes Assessments**

| **Diagnoses** | | **ICD9-CM** | **Used to Trigger Decision Support** |
| --- | --- | --- | --- |
| **Non-Antibiotic-Appropriate ARI Diagnoses**  **(Peer Comparison and Primary Outcome Assessment)** | | | |
|  | Acute nasopharyngitis (common cold) | 460 | non-specific URI |
|  | Acute laryngitis and tracheitis | 464, 464.0, 464.00, 464.1, 464.10, 464.2, 464.20, 464.4, 464.50 | non-specific URI |
|  | Acute laryngeopharyngitis/acute upper resp infection | 465, 465.0, 465.8, 465.9 | non-specific URI |
|  | Acute bronchitis | 466, 466.0, 466.1, 466.11, 466.19 | acute bronchitis |
|  | Bronchitis not specified as acute or chronic | 490 | acute bronchitis |
|  | Influenza | 487, 487.1, 487.8 | influenza |
| **Potentially-Antibiotic-Appropriate ARI Diagnoses**  **(Secondary Outcome Assessment)** | | | |
|  | Acute sinusitis | 461.xx | acute sinusitis/rhinosinusitis |
|  | Acute pharyngitis | 462 | acute pharyngitis |
|  |  |  |  |
| **Examples of Other ARIs Diagnoses or Symptoms of Interest**  **(Secondary Outcome Assessment)** | | | |
|  | Streptococal sore throat | 034.0 | acute pharyngitis |
|  | Cough | 786.2 | acute bronchitis |
|  | Pneumonia | 481-486 | none |

ARI: acute respiratory infection; ICD9-CM: International Classification of Diseases, Ninth Revision, Clinical Modification

**Additional file 1: Table S2. Results of Clinician Randomization and the Secondary Outcome Potentially-Antibiotic-Appropriate ARI Diagnoses**

| **Intervention** | **Randomized**  **Clinicians (n)** | **Antibiotic Prescribing for Visits with potentially-antibiotic-appropriate ARI diagnoses** | | **Difference in antibiotic prescribing rate between intervention and pre-intervention period**  **% (95% CI)** |
| --- | --- | --- | --- | --- |
|  |  | **Pre-Intervention year, n/N (%)** | **Intervention Year, n/N (%)** |  |
| No intervention | 4 | 47 / 84 (56.0) | 50 / 109 (45.9) | -10.1 (-24.4 to 4.2) |
| Accountable Justifications | 3 | 33 / 72 (45.8) | 33 / 70 (47.1) | 1.3 (-15.4 to 18.0) |
| Suggested Alternatives | 3 | 12 / 27 (44.4) | 10 / 25 (40.0) | -4.4 (-32.5 to 23.6) |
| Peer Comparisons | 3 | 31 / 65 (47.7) | 21 / 36 (58.3) | 10.6 (-10.1 to 31.3) |
| Accountable Justifications, Suggested Alternatives | 4 | 49 / 106 (46.2) | 10 / 47 (21.3) | -25.0 (-41.4 to -8.5) |
| Suggested Alternatives, Peer Comparisons | 4 | 39 / 99 (39.4) | 22 / 84 (26.2) | -13.2 (-26.9 to 0.5) |
| Accountable Justifications, Peer Comparisons | 4 | 152 / 264 (57.6) | 154 / 284 (54.2) | -3.4 (-11.7 to 5.0) |
| Accountable Justifications, Suggested Alternatives, Peer Comparisons | 3 | 48 / 100 (48.0) | 36 / 97 (37.1) | -10.9 (-24.8 to 3.0) |
| Any Accountable Justifications | 14 | 282 / 542 (52.0) | 233 / 498 (46.8) | -5.2 (-11.3 to 0.8) |
| No Accountable Justifications | 14 | 129 / 275 (46.9) | 103 / 254 (40.6) | -6.4 (-14.8 to 2.1) |
| Any Suggested Alternatives | 14 | 148 / 332 (44.6) | 78 / 253 (30.8) | -13.8 (-21.7 to -5.8) |
| No Suggested Alternatives | 14 | 263 / 485 (54.2) | 258 / 499 (51.7) | -2.5 (-8.8 to 3.7) |
| Any Peer Comparisons | 14 | 270 / 528 (51.1) | 233 / 501 (46.5) | -4.6 (-10.8 to 1.5) |
| No Peer Comparisons | 14 | 141 / 289 (48.8) | 103 / 251 (41.0) | -7.8 (-16.2 to 0.7) |
| All groups combined | 28 | 411 / 817 (50.3) | 336 / 752 (44.7) | -5.6 (-10.6 to -0.7) |

ARI: acute respiratory infection; CI: confidence interval

**Additional file 1: Table S3. Results of Clinician Randomization and the Secondary Outcome Other ARI Diagnoses or Symptoms of Interest**

| **Intervention** | **Randomized**  **Clinicians (n)** | **Antibiotic Prescribing for Visits with other ARIs diagnoses or symptoms of interest** | | **Difference in antibiotic prescribing rate between intervention and pre-intervention period**  **% (95% CI)** |
| --- | --- | --- | --- | --- |
|  |  | **Pre-Intervention year, n/N (%)** | **Intervention Year, n/N (%)** |  |
| No intervention | 4 | 26 / 120 (21.7) | 38 / 171 (22.2) | 0.6 (-9.2 to 10.3) |
| Accountable Justifications | 3 | 96 / 195 (49.2) | 45 / 116 (38.8) | -10.4 (-21.9 to 1.0) |
| Suggested Alternatives | 3 | 19 / 67 (28.4) | 5 / 55 (9.1) | -19.3 (-33.3 to -5.3) |
| Peer Comparisons | 3 | 64 / 113 (56.6) | 49 / 106 (46.2) | -10.4 (-23.7 to 2.9) |
| Accountable Justifications, Suggested Alternatives | 4 | 35 / 135 (25.9) | 21 / 117 (17.9) | -8.0 (-18.3 to 2.4) |
| Suggested Alternatives, Peer Comparisons | 4 | 184 / 374 (49.2) | 92 / 338 (27.2) | -22.0 (-29.0 to -15.0) |
| Accountable Justifications, Peer Comparisons | 4 | 140 / 377 (37.1) | 65 / 312 (20.8) | -16.3 (-23.1 to -9.5) |
| Accountable Justifications, Suggested Alternatives, Peer Comparisons | 3 | 82 / 227 (36.1) | 54 / 243 (22.2) | -13.9 (-22.1 to -5.8) |
| Any Accountable Justifications | 14 | 353 / 934 (37.8) | 185 / 788 (23.5) | -14.3 (-18.7 to -10.0) |
| No Accountable Justifications | 14 | 293 / 674 (43.5) | 184 / 670 (27.5) | -16.0 (-21.1 to -11.0) |
| Any Suggested Alternatives | 14 | 320 / 803 (39.9) | 172 / 753 (22.8) | -17.0 (-21.6 to -12.5) |
| No Suggested Alternatives | 14 | 326 / 805 (40.5) | 197 / 705 (27.9) | -12.6 (-17.3 to -7.8) |
| Any Peer Comparisons | 14 | 470 / 1091 (43.1) | 260 / 999 (26.0) | -17.1 (-21.1 to -13.0) |
| No Peer Comparisons | 14 | 176 / 517 (34.0) | 109 / 459 (23.7) | -10.3 (-16.0 to -4.6) |
| All groups combined | 28 | 646 / 1608 (40.2) | 369 / 1458 (25.3) | -14.9 (-18.2 to -11.6) |

ARI: acute respiratory infection; CI: confidence interval

**Additional file 1: Table S4. Results of Clinician Randomization and the Secondary Outcome All ARI Categories Combined**

| **Intervention** | **Randomized**  **Clinicians (n)** | **Antibiotic Prescribing for Visits with all ARI categories combined** | | **Difference in antibiotic prescribing rate between intervention and pre-intervention period**  **% (95% CI)** |
| --- | --- | --- | --- | --- |
|  |  | **Pre-Intervention year, n/N (%)** | **Intervention Year, n/N (%)** |  |
| No intervention | 4 | 80 / 261 (30.7) | 93 / 416 (22.4) | -8.3 (-15.2 to -1.4) |
| Accountable Justifications | 3 | 143 / 339 (42.2) | 86 / 256 (33.6) | -8.6 (-16.4 to -0.7) |
| Suggested Alternatives | 3 | 38 / 138 (27.5) | 19 / 131 (14.5) | -13.0 (-22.7 to -3.4) |
| Peer Comparisons | 3 | 126 / 252 (50.0) | 78 / 210 (37.1) | -12.9 (-21.9 to -3.8) |
| Accountable Justifications, Suggested Alternatives | 4 | 104 / 369 (28.2) | 33 / 259 (12.7) | -15.4 (-21.6 to -9.3) |
| Suggested Alternatives, Peer Comparisons | 4 | 264 / 591 (44.7) | 118 / 524 (22.5) | -22.2 (-27.5 to -16.8) |
| Accountable Justifications, Peer Comparisons | 4 | 336 / 828 (40.6) | 225 / 801 (28.1) | -12.5 (-17.1 to -7.9) |
| Accountable Justifications, Suggested Alternatives, Peer Comparisons | 3 | 176 / 498 (35.3) | 99 / 502 (19.7) | -15.6 (-21.1 to -10.2) |
| Any Accountable Justifications | 14 | 759 / 2034 (37.3) | 443 / 1818 (24.4) | -13.0 (-15.8 to -10.1) |
| No Accountable Justifications | 14 | 508 / 1242 (40.9) | 308 / 1281 (24.0) | -16.9 (-20.5 to -13.3) |
| Any Suggested Alternatives | 14 | 582 / 1596 (36.5) | 269 / 1416 (19.0) | -17.5 (-20.6 to -14.3) |
| No Suggested Alternatives | 14 | 685 / 1680 (40.8) | 482 / 1683 (28.6) | -12.1 (-15.3 to -8.9) |
| Any Peer Comparisons | 14 | 902 / 2169 (41.6) | 520 / 2037 (25.5) | -16.1 (-18.9 to -13.3) |
| No Peer Comparisons | 14 | 365 / 1107 (33.0) | 231 / 1062 (21.8) | -11.2 (-14.9 to -7.5) |
| All groups combined | 28 | 1267 / 3276 (38.7) | 751 / 3099 (24.2) | -14.4 (-16.7 to -12.2) |

ARI: acute respiratory infection; CI: confidence interval

**Additional file 1: Table S5. Distribution of Safety Monitoring Events**

| **Intervention Arm** | **Number of return visits where an antibiotic was not prescribed initially and may have been clinically useful had it been prescribed** |
| --- | --- |
|  |  |
| No intervention | 1 |
| Accountable Justifications | 1 |
| Suggested Alternatives | 1 |
| Peer Comparisons | 0 |
| Accountable Justifications, Suggested Alternatives | 1 |
| Suggested Alternatives, Peer Comparisons | 1 |
| Accountable Justifications, Peer Comparisons | 2 |
| Accountable Justifications, Suggested Alternatives, Peer Comparisons | 1 |
