# Supplementary material for: Behavioral interventions to reduce inappropriate antibiotic prescribing: a randomized pilot trial
Source: BMC Infect Dis. 2016 Aug 5;16:373. doi: 10.1186/s12879-016-1715-8 (PMC4975897; doi:10.1186/s12879-016-1715-8)
Supplement: Additional file 2: — Study Flow Diagram. (PPTX 77 kb) [file 12879_2016_1715_MOESM2_ESM.pptx]

## Slide 1
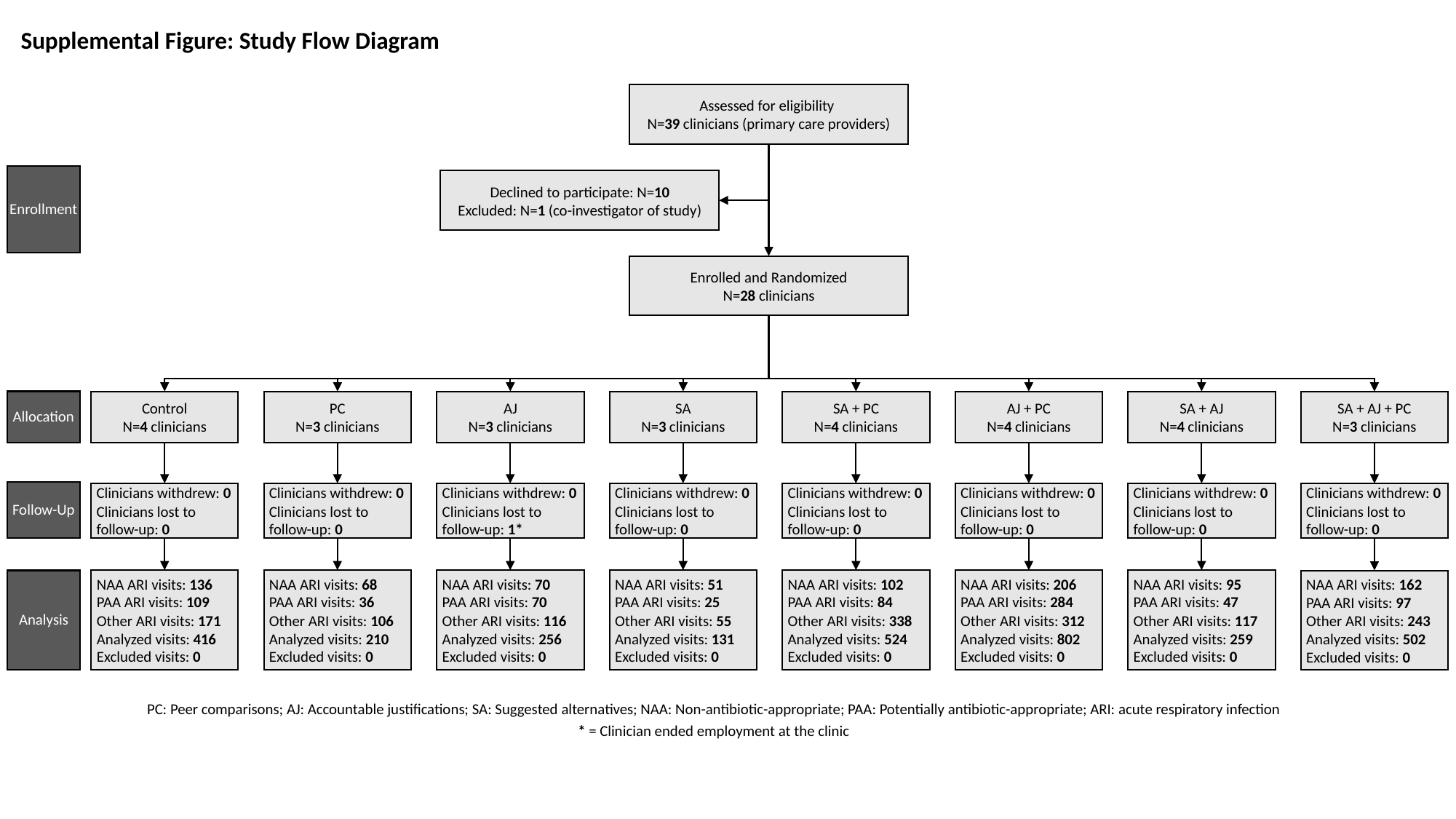

Supplemental Figure: Study Flow Diagram
Enrollment
Allocation
Follow-Up
Analysis
PC: Peer comparisons; AJ: Accountable justifications; SA: Suggested alternatives; NAA: Non-antibiotic-appropriate; PAA: Potentially antibiotic-appropriate; ARI: acute respiratory infection
* = Clinician ended employment at the clinic
